# MPPT of PEM Fuel Cell Using PI-PD Controller Based on Golden Jackal Optimization Algorithm

**DOI:** 10.3390/biomimetics8050426

**Published:** 2023-09-14

**Authors:** Ahmed M. Agwa, Tarek I. Alanazi, Habib Kraiem, Ezzeddine Touti, Abdulaziz Alanazi, Dhari K. Alanazi

**Affiliations:** 1 Department of Electrical Engineering, College of Engineering, Northern Border University, Arar 73222, Saudi Arabia; 2 Department of Electrical Engineering, Faculty of Engineering, Al-Azhar University, Cairo 11651, Egypt; 3 Department of Physics, College of Science, Northern Border University, Arar 73222, Saudi Arabia; 4 Processes, Energy, Environment and Electrical Systems, National Engineering School of Gabes, University of Gabes, Gabes 6029, Tunisia; 5 Electrical Engineering Department, Laboratory of Industrial Systems Engineering and Renewable Energies (LISIER), National Higher Engineering School of Tunis, Tunis 1008, Tunisia

**Keywords:** PEM fuel cell, MPPT, PI-PD controller, bioinspired algorithms, metaheuristic optimizers

## Abstract

Subversive environmental impacts and limited amounts of conventional forms of energy necessitate the utilization of renewable energies (REs). Unfortunately, REs such as solar and wind energies are intermittent, so they should be stored in other forms to be used during their absence. One of the finest storage techniques for REs is based on hydrogen generation via an electrolyzer during abundance, then electricity generation by fuel cell (FC) during their absence. With reference to the advantages of the proton exchange membrane fuel cell (PEM-FC), this is preferred over other kinds of FCs. The output power of the PEM-FC is not constant, since it depends on hydrogen pressure, cell temperature, and electric load. Therefore, a maximum power point tracking (MPPT) system should be utilized with PEM-FC. The techniques previously utilized have some disadvantages, such as slowness of response and largeness of each oscillation, overshoot and undershoot, so this article addresses an innovative MPPT for PEM-FC using a consecutive controller made up of proportional-integral (PI) and proportional-derivative (PD) controllers whose gains are tuned via the golden jackal optimization algorithm (GJOA). Simulation results when applying the GJOA-PI-PD controller for MPPT of PEM-FC reveal its advantages over other approaches according to quickness of response, smallness of oscillations, and tininess of overshoot and undershoot. The overshoot resulting using the GJOA-PI-PD controller for MPPT of PEM-FC is smaller than that of perturb and observe, GJOA-PID, and GJOA-FOPID controllers by 98.26%, 86.30%, and 89.07%, respectively. Additionally, the fitness function resulting when using the GJOA-PI-PD controller for MPPT of PEM-FC is smaller than that of the aforementioned approaches by 93.95%, 87.17%, and 87.97%, respectively.

## 1. Introduction

The replacement of traditional sources of energy based on fossil fuels with renewable energies (REs) is inevitable for environmental reasons and due to the gradual depletion of fossil fuels. REs are environmentally friendly and their sources are not exhaustible. The wind blows alternatingly so the wind speed varies continuously and in some cases is less than cut-in speed, i.e., the required speed to generate electrical energy. Similarly, solar energy is not available during the night and cloudy weather. Thus, the disadvantage of REs is that their sources, such as solar power and wind, are not available all the time. Consequently, REs should be stored to continually assure the existence of electrical energy [1,2]. A diversity of energy storage (ES) schemes exist that involve mechanical, magnetic, chemical, electrochemical, electrical, biological, and thermal energy storage. The choice of ES scheme relies considerably on the energy source, the energy required for special implementation, funds, and the viability of system infrastructure [3]. ES schemes involve:

- Mechanical ES: This includes ES in the form of kinetic, potential, or compression energy. The most frequently utilized schemes for ES as mechanical energy are flywheels and hydroelectric pump storage [4]. Other mechanical ES schemes exist, such as springs, compressed air, hydraulic accumulators, and gravitational potential.
- Magnetic ES: In this scheme, ES is performed through supplying DC current via a coil and creating a magnetic field. In most circumstances, a superconducting magnetic coil is employed [5]. The cooling process of the superconducting magnet can release the stored energy once again into the surroundings.
- Chemical ES: In this scheme, ES is performed via chemical or physical suction, intercalation, electrochemical procedures, or chemical conversion [6]. Presently, there is increased interest in employing methanol, methane, butanol, hydrogen, and hydrocarbons for chemical ES schemes [7].
- Electrical ES: In this scheme, ES in the form of electrical charge is performed, i.e., obtained via electricity; this process is generally accomplished via capacitors or supercapacitors [8].
- Biological ES: These schemes in general store energy which has been produced through breakdown of glucose via enzymes [9]; nevertheless, an obstacle to biological ES schemes is that their efficiency is low at about 10%.
- Thermal ES: In this scheme, ES is performed via storing heat in a latent, sensible or absorption manner. These schemes provide good opportunities for waste heat recovery and for domestic cooling/heating techniques [10].
- Electrochemical ES: The storage of electrical energy generated via RE sources in the form of electrochemical energy using rechargeable batteries is commonly implemented. Unfortunately, the life span of rechargeable batteries is short, so they need to be continuously replaced, which adds to their cost. Fuel cells (FCs) are promising means for extracting the stored energy via intermittent REs in the absence of a combustion process [11]. Hydrogen is produced by the surplus REs via electrolyzers then, when there is shortage in REs, electrical energy is generated from hydrogen by FCs [2]. Because of the merits of the proton exchange membrane fuel cell (PEM-FC), it is favored over other types of FC. The optimum operation of PEM-FC is investigated in this article.

For each group of operation conditions for PEM-FC, i.e., hydrogen pressure, cell temperature, and electric load, there exists a unique point on the current–power (I/P) plot representing maximum power. Accordingly, the maximum power point tracking (MPPT) procedure is required for extraction of the maximum power from the PEM-FC at various operation conditions. The MPPT system is a DC-DC boost converter with an adjustable duty switch cycle (DU). The DC-DC boost converter is fed via the stack terminal voltage (V_sta_) of PEM-FC and DU is adjusted to make the output voltage (V_o_) track the voltage at MPP (V_MP_). The difference between the various approaches to MPPT by PEM-FC is the determination method of V_MP_ for adjustment of DU via the DC-DC boost converter.

In this regard, perturb and observe (P&O) [12,13] has been utilized for MPPT of PEM-FC, where V_o_ is repeatedly varied via varying DU by a fixed step (ΔDU), the resultant power and voltage variations are observed and, accordingly, DU is increased or decreased in the next variation until reaching MPP. The authors of [14] have utilized fuzzy logic (FL) to determine ΔDU size of P&O. In the incremental conductance (IC) [15] and the incremental resistance (IR) [16] methods, V_MP_ is determined wherever the derivative of power of the PEM-FC stack $\left(P_{sta}\right)$ with regards to the operating current of the FC ($I_{fc}$) equals zero. The authors of [17,18] have employed a backstepping technique to determine DU, which makes I_FC_ track the current at MPP. In the prementioned methods, the power of PEM-FC is calculated via multiplying the measured values of V_o_ and I_FC_ while, in the variable step size (VSS) [19,20,21], the measured value of I_FC_ is only utilized to decrease the cost and complexity.

In [22,23,24,25], V_sta_ and I_FC_ are entered into the trained artificial neural network (ANN) to produce the DU of the DC-DC boost converter. Adaptive ANN based on a fuzzy inference system has been applied in [26,27,28].

The authors of [29,30,31] have employed FL for determination of DU of the DC-DC boost converter for MPPT of PEM-FC. In the same regard, several controllers have been employed, such as model predictive control (MPC) [32], sliding mode controller (SMC) [33,34,35], fuzzy logic controller (FLC) [36,37,38,39,40], FLC-based VSS [41,42,43], and FLC optimized by various algorithms, e.g., firefly optimizer [44] and differential evolution (DE) [45].

In addition to the aforementioned controllers, numerous others have been employed for MPPT of PEM-FC, i.e., the proportional-integral-derivative (PID) controller optimized by numerous algorithms, such as salp swarm approach (SSA) [46], the particle swarm optimizer(PSO) [47], the grey wolf optimizer (GWO) [48], the fractional-order PID controller optimized by forensic-based investigation optimizer [49], and the fractional-order integral controller with filter [50].

Table 1 summarizes the limitations of many published techniques. This article addresses the deficiencies of the former published works by proposing an innovative PI-PD controller for MPPT of PEM-FC. The gains of the PI-PD controller are adjusted through the golden jackal optimization algorithm (GJOA). The suggested approach (GJOA-PI-PD) and controller has the potential for improving results, since its structure (PI-PD controller) is different from those in the literature, i.e., the PID and FOPID controllers. Additionally, the results of applying the PI-PD controller for automatic generation control in [51] revealed its advantages over PID and PI controllers.

Recently, metaheuristic optimization techniques have been applied for numerous purposes. Three kinds of these techniques are employed: evolutionary algorithms, physics-based, and swarm intelligence techniques. The first kind is driven by relying on biological evolution, e.g., DE and artificial bee colony. The second kind is driven by relying on physical laws, e.g., equilibrium algorithm and Archimedes optimizer. The last kind is driven by relying on the manners of animal groups, e.g., PSO, SSA, and GWO.

In this regard, the GJOA is suggested for adjusting the gains of the PI-PD controller. The GJOA is a metaheuristic optimizer that replicates the golden jackal’s manner during hunting [52]. GJOA was written in 2022 and utilized successfully for economic dispatch [52], planning of wind turbines, and for charging stations of electric vehicles [53]. The successful utilization of GJOA for engineering optimization issues encouraged the authors to employ it to adjust the gains of the PI-PD controller for MPPT of PEM-FC.

The contributions of this article are:

1. The innovative employment of the PI-PD controller for MPPT of PEM-FC.
2. The innovative application of GJOA for adjustment of the gains of the PI-PD controller.
3. Comparing the acquired results using the GJOA-PI-PD controller for MPPT of PEM-FC with those based on the P&O approach, GJOA-PID, and GJOA-FOPID controllers in order to confirm its supremacy.
4. The GJOA-PI-PD controller performance is validated through variations in hydrogen pressure, cell temperature, and electric load.

The remainder of the article is organized as follows: FCs are overviewed in Section 2. The PEM-FC is modeled in Section 3. The DC-DC boost converter is revealed in Section 4. The proposed control strategy of MPPT is clarified in Section 5, including the PI-PD controller and GJOA plus the compared control strategies i.e., FOPID controller and P&O. The results are discussed in Section 6. Conclusions are extracted in Section 7.

## 2. Overview of FCs

**Figure 1:**
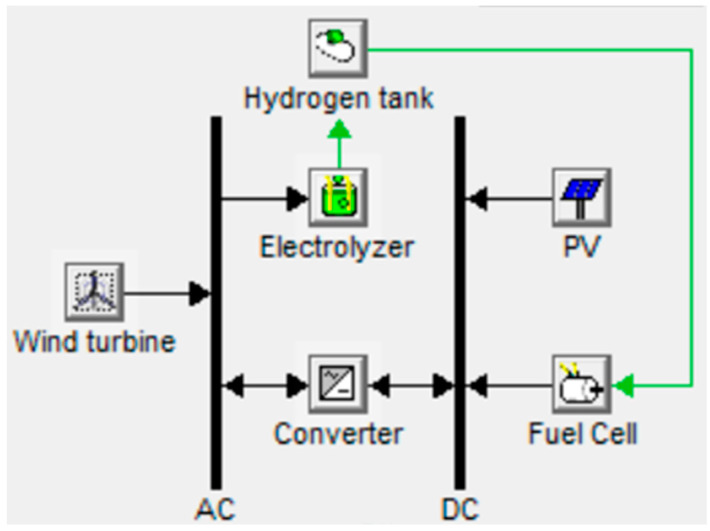
Complete scheme of FC utilized for electrochemical ES of REs.

Figure 1 reveals the complete scheme of the FC utilized for electrochemical ES of REs. The water is distilled, then supplied to the electrolyzer by water pump. The electrolyzer produces one molecule of hydrogen (H_2_) from each molecule of water (H_2_O). The relationship between electrical power ($P_{e}$) and the volumetric hydrogen rate (${\dot{V}}_{He}$) (m^3^/h) is stated in (1) [54]:

(1) $$\eta_{e}=\frac{P_{e}}{{\dot{V}}_{He}}=\frac{1}{3600}\cdot \frac{\rho_{H}\cdot e\cdot F\cdot U_{rev}}{\eta_{I}{\cdot \eta }_{U}\cdot M_{r}}$$

where $\eta_{e},\eta_{I},$ and $\eta_{U}$ are electrical, current, and voltaic efficiencies, respectively, $\rho_{H}$ is the hydrogen density (0.08988 kg/m^3^), e is the number of electrons implied in the reaction and equals 2 for water splitting, F is Faraday’s number (96,485 As/mol), $U_{rev}$ is the reversible cell voltage, and $M_{r}$ is the relative molecular mass (2.016 g/mol). The reversible PEM fuel cells exhibited a round-trip electrical efficiency of 40–46% at current density of 500 mA/cm^2^. The energy conversion process inside is clean, since FC exhaust is water vapor.

FCs are mainly categorized according to their electrolyte. This categorization establishes the type of electrochemical reactions which occur inside the FC, the type of catalysts needed, the temperature limit of the FC, the fuel needed, and other features. These FC characteristics impact their appropriate purposes. The kinds of FC include proton exchange membrane fuel cell (PEM-FC), solid oxide FC, phosphoric acid FC, alkaline FC, molten FC, and direct methanol [55]. Comparison among kinds of FCs reveals that PEM-FC is distinguished by its low operating temperature, great power extent, rapid start-up, little corrosion, simple composition, light weight, small volume, cheap cost, and long life span [56]. Additionally, the solid electrolyte of PEM-FCs makes electrode sealing simpler than with other kinds of FC. The operating temperature of PEM-FC ranges between 60 and 100 °C. The entire expense of a car based on the PEM-FC is 500–600 $/kW [57]. Thus, PEM-FCs are employed in several applications for instance transportation [56], airplanes, and distributed generators [58].

## 3. PEM-FC Model

The PEM-FC stack model has been densely illustrated in the literature. For a stack composed of $n_{cells}$ as a series connected cells, $V_{sta}$ can be calculated as below [19,21]:

(2) $$V_{sta}=n_{cells}\cdot \left(E-V_{acti}-V_{con}-V_{\Omega }\right)$$

where E is open circuit potential, $V_{acti}$ and $V_{con}$ are activation and concentration over-voltages for each cell, respectively, and $V_{\Omega }$ is ohmic voltage drop for each cell. These variables are computed using (3) to (6) [19,21].

(3) $$E=1.299-0.85\cdot 10^{-3}\left(T_{fc}-298.15\right)+4.3085\cdot 10^{-5}T_{fc}ln\left(P_{H_{2}}\sqrt{P_{O_{2}}}\right)$$

where $T_{fc}$ is cell temperature (K), and $P_{O_{2}}$ and $P_{H_{2}}$ are partial pressures $\left(atm\right)$ of $O_{2}$ and $H_{2}$, respectively.

(4) $$V_{act}=-\left[\xi_{1}+\xi_{2}T_{fc}+\xi_{3}T_{fc}ln\left(C_{O_{2}}\right)+\xi_{4}T_{fc}ln(I_{fc})\right]$$

where $\xi_{i}\left(i\in \left\{1,2,3,4\right\}\right)$ are empirical parameters, $C_{O_{2}}$ is concentration of $O_{2}(mol/{cm}^{3})$, and $I_{fc}$ is the operating current (A) of the FC.

(5) $$V_{con}=-b\cdot ln\left(\frac{J_{max}-J}{J_{max}}\right)$$

where b is parametric coefficient, and J and $J_{max}$ are actual and maximum density of current $\left(A/{cm}^{2}\right)$, respectively.

(6) $$V_{Ω}=I_{fc}\left(R_{m}+R_{C}\right)$$

where $R_{m}$ and $R_{C}$ are resistances (Ω) of the membrane and connections, respectively.

$P_{sta}$ is computed as below:

(7) $$P_{sta}=V_{sta}\cdot I_{fc}$$

**Figure 2:**
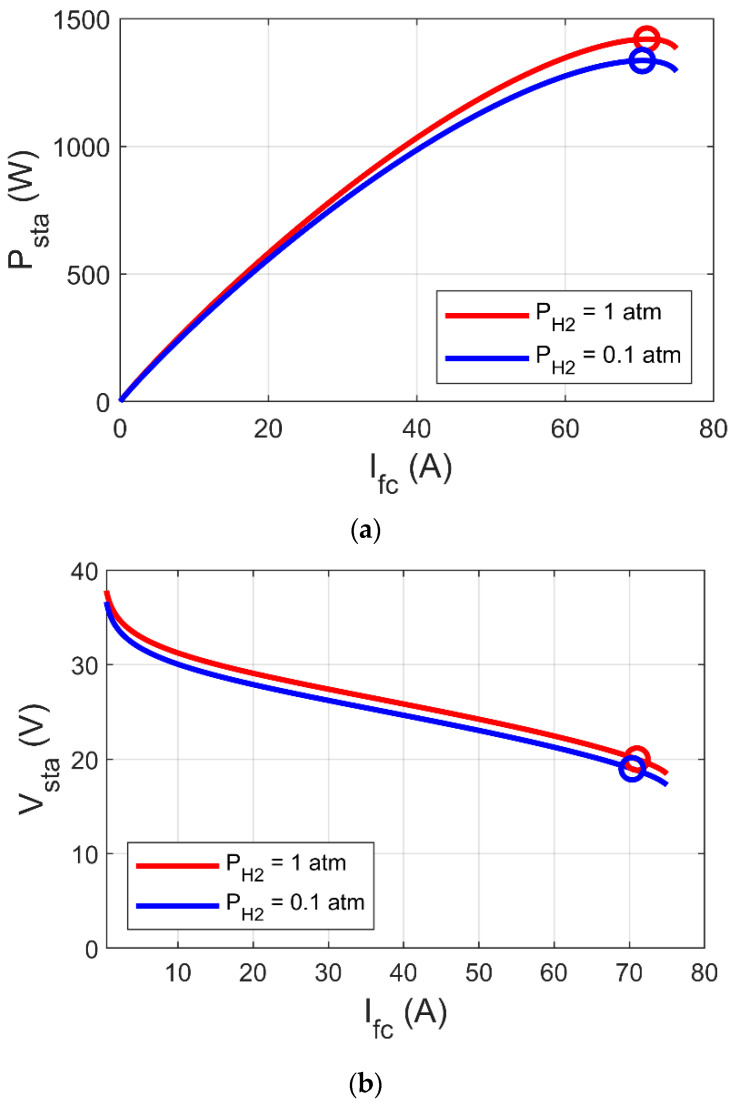
Impact of $P_{H_{2}}$ variations on MPP of PEM-FC. (**a**) I/P cs. (**b**) I/V cs.

**Figure 3:**
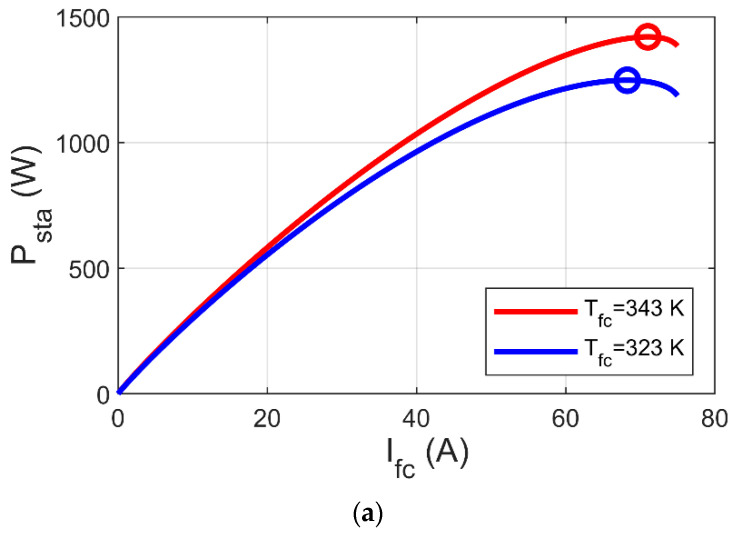

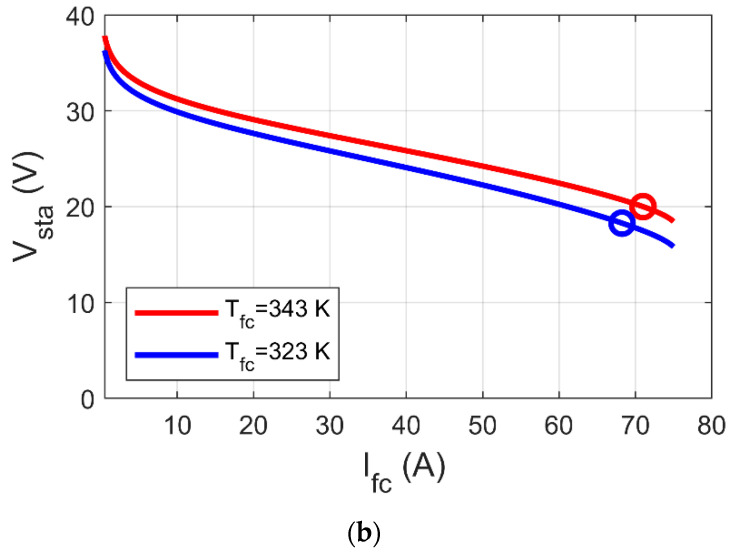
Impact of $T_{fc}$ variations on MPP of PEM-FC. (**a**) I/P cs. (**b**) I/V cs.

By reference to $\left(2\right)$ to $\left(7\right)$, it is clear that $P_{sta}$ is reliant on $P_{H_{2}}$, $T_{fc}$, and $I_{fc}$ which is reliant on electric load. Figure 2 and Figure 3 show the variation of MPP based on the variations of $P_{H_{2}}$ and $T_{fc}$, respectively, where MPP increases with increase of both $P_{H_{2}}$ and $T_{fc}$. In Figure 2b and Figure 3b, it can be observed that MPP occurs at a specific voltage (V_MP_), which is reliant on $P_{H_{2}}$, $T_{fc}$, and electric load. Thus, the key to reach MPP is to raise V_fc_ to V_MP_ using the DC-DC boost converter. In this article, we suggest an innovative MPPT for PEM-FC using the PI-PD controller, whose gains are tuned by GJOA. We begin with an explanation of the DC-DC boost converter in the next section.

## 4. DC-DC Boost Converter

**Figure 4:**
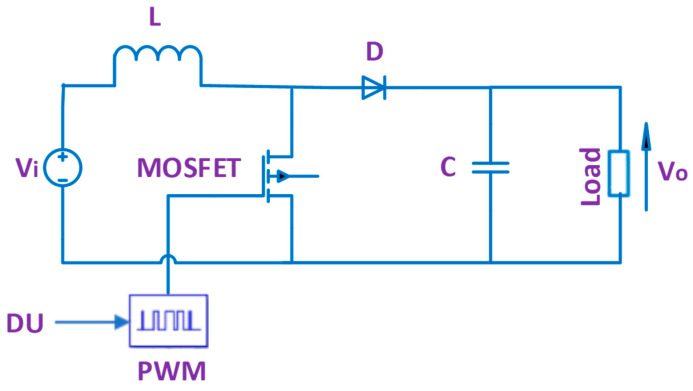
DC-DC boost converter.

Figure 4 reveals the DC-DC boost converter, comprised of an inductor (L) to store energy, MOSFET to switch on and off, a diode (D) to insulate between the input and output intervals, and a capacitor (C) to lessen ripples. Additionally, a pulse width modulator (PWM) supplies pulses to the gate of MOSFET [17,26,28,33,40,48]. The width of pulses is modulated depending on DU. The number of pulses per second is determined via switching frequency (f_swi_) of PWM.

V_o_ is dependent on input voltage (V_i_) and DU as stated in (8) [17,26,28,33,40,48].

(8) $$V_{o}=\frac{1}{1-DU}\cdot V_{i}$$

For known values of V_i_ and V_o_, the value of DU can be derived from (8) as below:

(9) $$DU=1-\frac{V_{i}}{V_{o}}$$

When the DC-DC boost converter is employed for MPPT of PEM-FC, V_sta_ and V_MP_ represent V_i_ and V_o_, respectively. Since V_sta_ and V_MP_ change continuously, then DU needs to be adjusted continuously. The suggested control strategy for adjusting the DU of the DC-DC boost converter for MPPT of PEM-FC is illustrated in the next section.

## 5. MPPT Control Strategy

**Figure 5:**
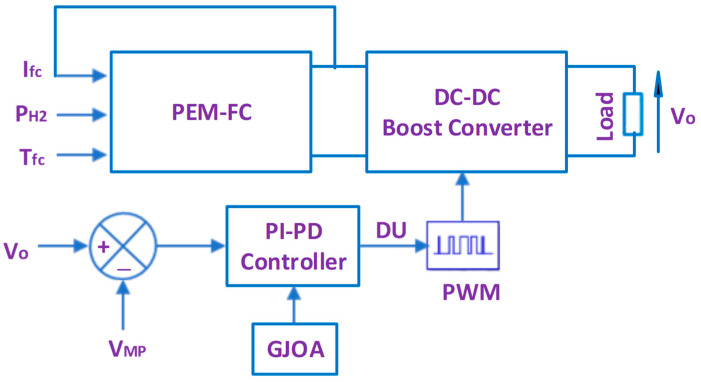
Schematic diagram of the suggested MPPT control scheme.

Figure 5 reveals the schematic diagram of the suggested control scheme for MPPT of the PEM-FC, where DU of the DC-DC boost converter is tuned using a PI-PD controller whose gains are tuned using GJOA. The input of the PI-PD controller is the difference between V_MP_ and V_o_ in order to make V_o_ track V_MP_ continuously, and then $P_{sta}$ tracks MPP continuously. The details of the PI-PD controller, GJOA, and fitness function (FiFu) of the GJOA are illustrated in Section 5.1, Section 5.2 and Section 5.3, respectively. Afterwards the compared approaches, i.e., FOPID controller and P&O, are illustrated in Section 5.4 and Section 5.5, respectively.

### 5.1. PI-PD Controller

**Figure 6:**
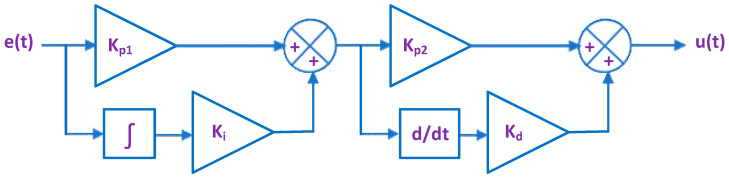
PI-PD controller.

Figure 6 reveals that the PI-PD controller is a consecutive controller made up of PI and PD controllers whose gains are $K_{p_{1}}$, $K_{i}$, $K_{p_{2}}$, and $K_{d}$, respectively. The mathematical relationship between the output control signal (u(t)) and the input error signal (e(t)) of the PI-PD controller is stated in (10).

(10) $$u\left(t\right)=K_{p_{2}}\cdot \left(K_{p_{1}}\cdot e\left(t\right)+K_{i}\cdot \int e\left(t\right)\cdot dt\right)+K_{d}\cdot \frac{d}{dt}\left(K_{p_{1}}\cdot e\left(t\right)+K_{i}\cdot \int e\left(t\right)\cdot dt\right)$$

### 5.2. GJOA

The GJOA is a swarm intelligence optimizer which imitates the hunting manner of golden jackals in wildlife. Their hunting group consists of females and males. There are three stages in their hunting manner: 1—seeking and approaching the prey; 2—surrounding and confusing the prey, stopping its movement; 3—swooping on the prey.

Throughout the initialization step, a group of prey locations matrix ($Prey\left(0\right)$) is produced randomly using (11) [52].

(11) $$Prey\left(0\right)=\left[\begin{array}{ccc} \begin{array}{cc} L_{1,1} & L_{1,2}\\ L_{2,1} & L_{2,2} \end{array} & \cdots & \begin{array}{c} L_{1,dim}\\ L_{2,dim} \end{array}\\ \vdots & \ddots & \vdots \\ \begin{array}{cc} L_{pop,1} & L_{pop,2} \end{array} & \cdots & L_{pop,dim} \end{array}\right]$$

where pop symbolizes the population of the prey and dim symbolizes dimension.

E is the escaping energy of the prey and is computed using (11) [53].

(12) $$E=E_{1}\cdot E_{0}$$

where $E_{1}$ and $E_{0}$ indicate the diminishing energy of the prey and the initial energy, respectively. Value of $E_{0}$ ranges from [−1, 1], while the value of $E_{1}$ is computed via (13) [52].

(13) $$E_{1}=c_{1}\cdot \left(1-\frac{ite}{max\_ite}\right)$$

where $c_{1}$ represents a fixed number, whose value is 1.5, ite represents the current iteration, and max_ite represents maximum number of iterations.

If $\left|E\right|>1$, golden jackal hunting is mathematically modelled using (14) and (15) [53]:

(14) $$L_{1}\left(ite\right)=L_{M}\left(ite\right)-E\cdot \left|L_{M}\left(ite\right)-rl\cdot Prey\left(ite\right)\right|$$

(15) $$L_{2}\left(ite\right)=L_{FM}\left(ite\right)-E\cdot \left|L_{FM}\left(ite\right)-rl\cdot Prey\left(ite\right)\right|$$

where $L_{1}\left(ite\right)$ and $L_{2}\left(ite\right)$ are the updated locations of the male and female jackals, respectively, $L_{M}\left(ite\right)$ and $L_{FM}\left(ite\right)$ symbolize the locations of the male and the female jackals, respectively, rl symbolizes the vector of numbers calculated randomly via the Levy flight function, $Prey\left(ite\right)$ symbolizes the vector of prey locations, $\left|L_{M}\left(ite\right)-rl\cdot Prey\left(ite\right)\right|$ represents the spacing among the prey and the jackal, and rl symbolizes a vector of numbers calculated randomly via the Levy flight function as stated in (16) and (17) [52].

(16) $$rl=0.05\cdot Lf\left(z\right)$$

(17) $$Lf\left(z\right)=\frac{0.01\cdot u\cdot \sigma }{\left|v^{\left(\frac{1}{\beta }\right)}\right|};\sigma ={\left[\frac{\Gamma \left(1+\beta \right)\cdot \sin\frac{\pi \cdot \beta }{2}}{\Gamma \left(\frac{1+\beta }{2}\right)\cdot \beta \cdot \left(2^{\beta -1}\right)}\right]}^{\frac{1}{\beta }}$$

where u and v are randomly determined values between 0 and 1 and β symbolizes a fixed number whose value is 1.5.

As the prey is fatigued due to the chase, E is diminished and meanwhile, when $\left|E\right|\leq 1$, the jackals surrounding the prey and gobbling it up are mathematically modelled using (18) and (19) [53]:

(18) $$L_{1}\left(ite\right)=L_{M}\left(ite\right)-E\cdot \left|rl\cdot L_{M}\left(ite\right)-Prey\left(ite\right)\right|$$

(19) $$L_{2}\left(ite\right)=L_{FM}\left(ite\right)-E\cdot \left|{rl\cdot L}_{FM}\left(ite\right)-Prey\left(ite\right)\right|$$

The updated location of the prey $\left(L\left(ite+1\right)\right)$ is computed via the average of $L_{1}\left(ite\right)$ and $L_{2}\left(ite\right)$ as stated in (20) [52].

(20) $$L\left(ite+1\right)=\frac{L_{1}\left(ite\right)+L_{2}\left(ite\right)}{2}$$

**Figure 7:**
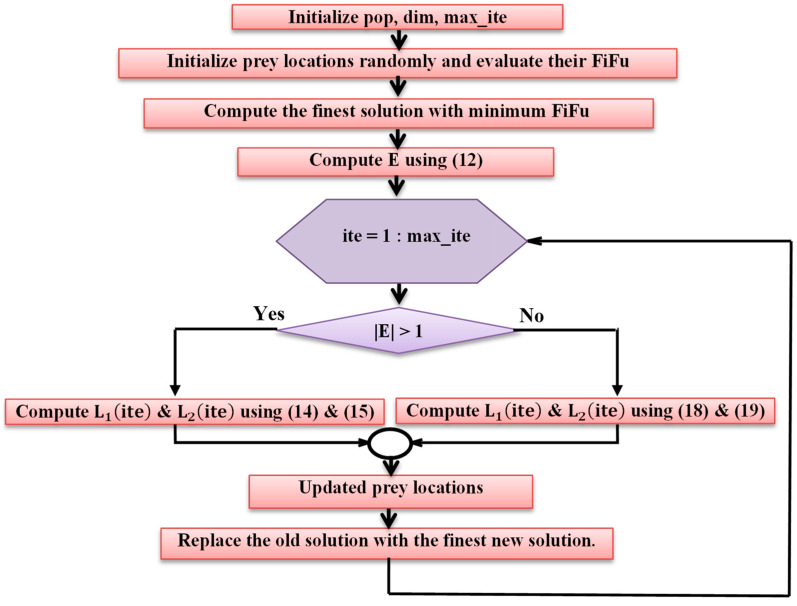
Flowchart of GJOA.

All details of the GJOA can be found in [52]. The MATLAB code of GJOA can be found in [59]. Figure 7 reveals the flowchart of GJOA.

### 5.3. Formularization of FiFu

In this subsection, we formulate the FiFu to be minimized by GJOA while tuning the gains of PI-PD controller, which in turn adjusts the DU of the DC-DC boost converter for MPPT of PEM-FC.

The main aim of MPPT of PEM-FC is to make P_sta_ track MPP quickly with minimal oscillations and overshoot, throughout the variations in $P_{H_{2}}$, $T_{fc}$, or load. To achieve this, the DC-DC boost converter is utilized to make $V_{o}$ track $V_{MP}$ as rapidly as possible. The requirements mentioned are guaranteed using the integral-time-absolute errors (ITAE) of $\left|\left(V_{o}-V_{MP}\right)\right|$ as a superior criterion compared to other criteria, such as integral absolute error, integral square error, and integral time square error, as proven in [60]. Minimization of ITAE results in decrease of response time, overshoot, and oscillations, hence FiFu is proposed to minimize the ITAE, as stated below [60]:

(21) $$FiFu=minimize\left(ITAE\right)=minimize\left(\int_{0}^{t_{si}}t\cdot \left|\left(V_{o}-V_{MP}\right)\right|\cdot dt\right)$$

where $t_{si}$ symbolizes the simulation time. The FiFu is subjugated via constraints to maintain the gains of the PI-PD controller within predefined limits.

### 5.4. FOPID Controller

The FOPID controller differs from the PID controller in that the order of both integration and differentiation is a fraction instead of an integer. The transfer function of the FOPID controller is stated in (22) [49].

(22) $$C\left(s\right)=K_{p}+\frac{K_{i}}{s^{\gamma }}+K_{d}s^{\mu }$$

where $K_{p}$, $K_{i}$, and $K_{d}$ symbolize the gains of FOPID controller and λ and µ symbolize the order of integration and differentiation, respectively.

### 5.5. P&O

**Figure 8:**
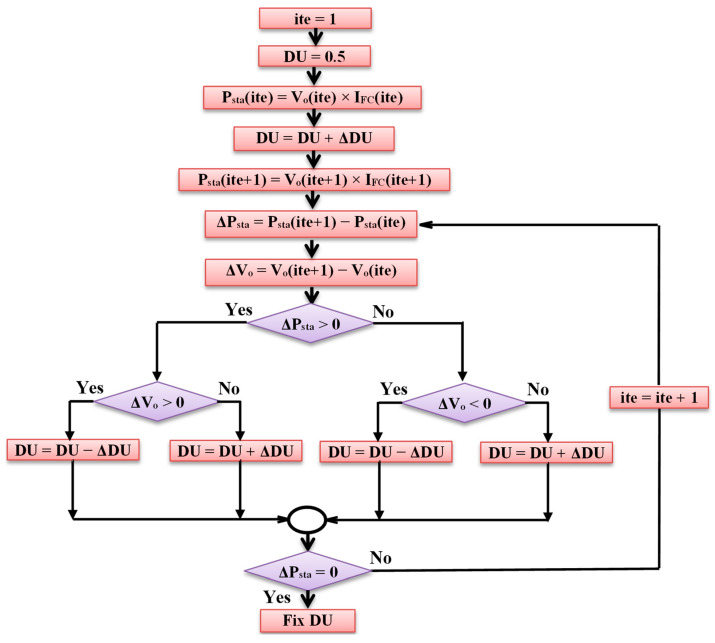
Flowchart of P&O.

P&O is an iterative approach for MPPT of PEM-FC. P&O is commonly utilized for its simplicity. Firstly, V_o_ and I_FC_ are measured and P_sta_ is calculated via multiplying their values, then DU is changed by fixed ΔDU, which leads to a change in V_o_ and, accordingly, P_sta_. The resultant changes in P_sta_ (ΔP_sta_) and V_o_ (ΔV_o_) are monitored. If ΔP_sta_ and ΔV_o_ have the same sign, then DU is decreased by ΔDU, otherwise DU is increased by ΔDU in the next iteration. This procedure is repeated until ΔP_sta_ equals zero, i.e., it reaches MPP. Figure 8 reveals the flow chart of the P&O approach [13].

## 6. Results with Discussion

The efficacy and forcefulness of MPPT of PEM-FC based on the GJOA-PI-PD controller are endorsed via comparing its results with those of other approaches. The impact of variations in $P_{H_{2}}$, $T_{fc}$, and loading on the performance of the suggested MPPT of PEM-FC is also examined.

The simulation results have been obtained via MATLAB-R2021 in Windows 11.

**Figure 9:**
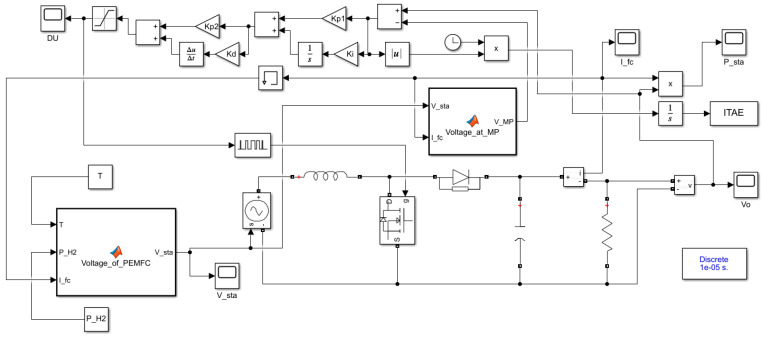
Simulink model of the suggested MPPT for PEM-FC.

The GJOA is operated with these parameters: pop = 10 and max_ite = 5. The MPPT is performed on a commercial typical PEM-FC, namely the Ballard Mark V, whose parameters are listed in Table 2. These parameter values were extracted using the whale optimizer in [61]. Regarding the values of parameters of the DC-DC boost converter, f_swi_ = 10 kHz, high f_swi_ is chosen to downsize the capacitors and inductors, which causes a cost decrease, L = 69 mH, and C = 1500 μF. These settings of L and C are carefully selected to assure low ripples in V_o_ at the indicated f_swi_. The limits within which the parameters of GJOA-PID, GJOA-FOPID, and GJOA-PI-PD controllers are maintained during minimization of FiFu using GJOA are listed in Table 3, Table 4 and Table 5, respectively. Figure 9 reveals the MATLAB Simulink model of the suggested MPPT for PEM-FC.

### 6.1. MPPT of PEM-FC under Normal Operating Conditions

Normal operating conditions of $P_{H_{2}}$ and $T_{fc}$ for the Ballard Mark V PEM-FC are applied in this case for different schemes of MPPT of PEM-FC. In detail, $P_{H_{2}}=1\;atm$ and $T_{fc}=343\;K$. Regarding the electric load, resistance (R) of 50 Ω is supplied by PEM-FC.

The values of optimized parameters of GJOA-PID, GJOA-FOPID, and GJOA-PI-PD controllers are listed in Table 6, Table 7 and Table 8, respectively.

**Figure 10:**
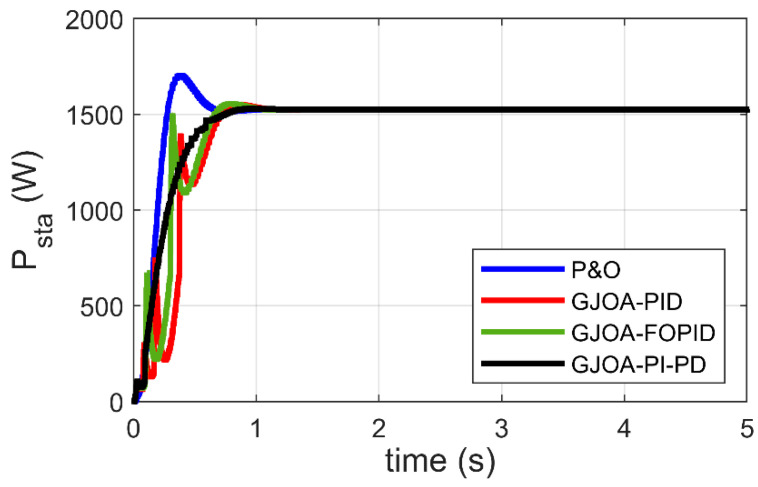
Response of MPPT schemes for Ballard Mark V PEM-FC.

Figure 10 reveals P_sta_ of the Ballard Mark V PEM-FC when three MPPT schemes, plus the proposed scheme, are applied. Specifically, the P&O approach, GJOA-PID, and GJOA-FOPID controllers are compared with the proposed GJOA-PI-PD controller. High overshoot exists in the response of P_sta_ when the P&O scheme is employed. There are oscillations and slowness in the response of P_sta_ when GJOA-PID, and GJOA-FOPID controllers are employed. The resultant values of rise time (t_r_) and percentage overshoot (POS) for various MPPT schemes are listed in Table 9. The proposed GJOA-PI-PD controller results in POS of 0.2% which is the lowest overshoot compared to other MPPT schemes i.e., the P&O approach, GJOA-PID, and GJOA-FOPID controllers, by 98.26%, 86.30%, and 89.07%, respectively. The resultant value of t_r_ with the proposed GJOA-PI-PD controller is 0.391 s, which is less than that of the GJOA-PID, and GJOA-FOPID controllers but more than that of P&O. The criteria in comparison are that the MPPT scheme, which has the quickest response, the least oscillations, and the lowest overshoot, is preferred over other schemes. When these criteria are applied to the results revealed in Table 8, the proposed GJOA-PI-PD controller is found to have better equilibrium among speed and overshoot than other MPPT schemes.

The previous comparison is based on visual analysis of the results. On the other hand, the comparison based on the numerical results of ITAE confirms the preference for the GJOA-PI-PD controller over other schemes, as summarized in Table 10, where the values of ITAE are listed. The value of ITAE resulting from the GJOA-PI-PD controller is the least compared to the others, by 93.95%, 87.17%, and 87.97%. It can be said that MPPT based on the GJOA-PI-PD controller outperforms other approaches by a wide margin. The MPPT schemes can be arranged in accordance with the smallness of ITAE as follows: GJOA-PI-PD, GJOA-PID, GJOA-FOPID controllers, then the P&O scheme.

### 6.2. MPPT of PEM-FC under Variation of $P_{H_{2}}$

**Figure 11:**
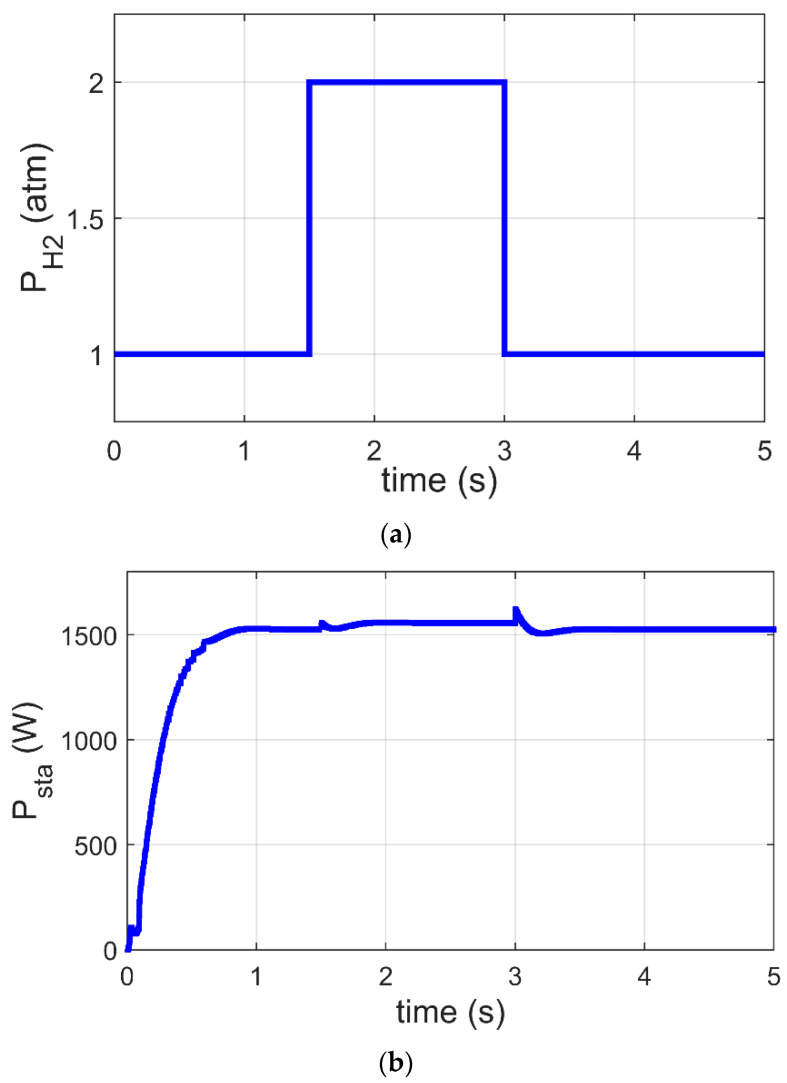
Response of MPPT based on GJOA-PI-PD controller at variation of $P_{H_{2}}$. (**a**) Variation of $P_{H_{2}}$. (**b**) P_sta_ of Ballard Mark V PEM-FC.

In this subsection, the GJOA-PI-PD controller for MPPT of the Ballard Mark V PEM-FC is validated when $P_{H_{2}}$ changes. Figure 11a reveals that the value of $P_{H_{2}}$ is initially 1 atm, then it increases to 2 atm at t=1.5 s, and afterward it decreases to 1 atm at t=3 s. Figure 11b reveals the corresponding response of P_sta_ during a change in $P_{H_{2}}$ where we observe that MPPT based on the GJOA-PI-PD controller reacts speedily to variation in $P_{H_{2}}$. During the period of increase of $P_{H_{2}}$, P_sta_ increases to new value then decreases with decrease of $P_{H_{2}}$. This means that P_sta_ tracks the new MPP for new conditions. The new conditions in this case study resulted in a variation of $P_{H_{2}}$ from 1 atm to 2 atm and then from 2 atm to 1 atm, with constant values of $T_{fc}=343\;K$ and R=50 Ω. Additionally, the absence of oscillations is observed. Furthermore, the values of overshoot and undershoot are very small.

### 6.3. MPPT of PEM-FC under Variation of $T_{fc}$

**Figure 12:**
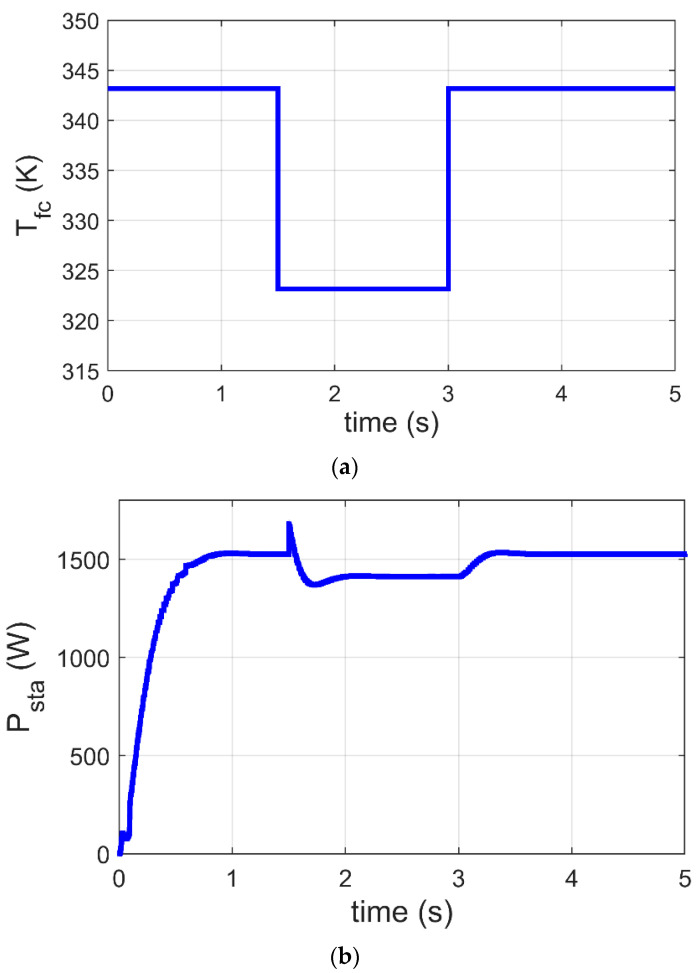
Response of MPPT based on the GJOA-PI-PD controller at variation of $T_{fc}$. (**a**) Variation of $T_{fc}$. (**b**) P_sta_ of Ballard Mark V PEM-FC.

This part presents a justification for the GJOA-PI-PD controller for MPPT of the Ballard Mark V PEM-FC when $T_{fc}$ varies. The change in $T_{fc}$ is revealed in Figure 12a, where it is initially 343 K, then it decreases to 323 K at t=1.5 s, and after that it increases to 343 K at t=3 s. The corresponding response of P_sta_ during variation of $T_{fc}\;$is illustrated in Figure 12b, where the quick performance of MPPT based on the GJOA-PI-PD controller with variation of $T_{fc}$ is observed. Throughout the period of decrease in $T_{fc}$, P_sta_ decreases to its new value then increases with increase in $T_{fc}$. This indicates that P_sta_ tracks new MPP for new conditions. The new conditions in this case study are caused by change in $T_{fc}$ from 343 K to 323 K and then from 323 K to 343 K, with constant values of $P_{H_{2}}=1\;atm$ and R=50 Ω. Moreover, there are no high values for oscillations during variation in P_sta_.

### 6.4. MPPT of PEM-FC under Variation of R

**Figure 13:**
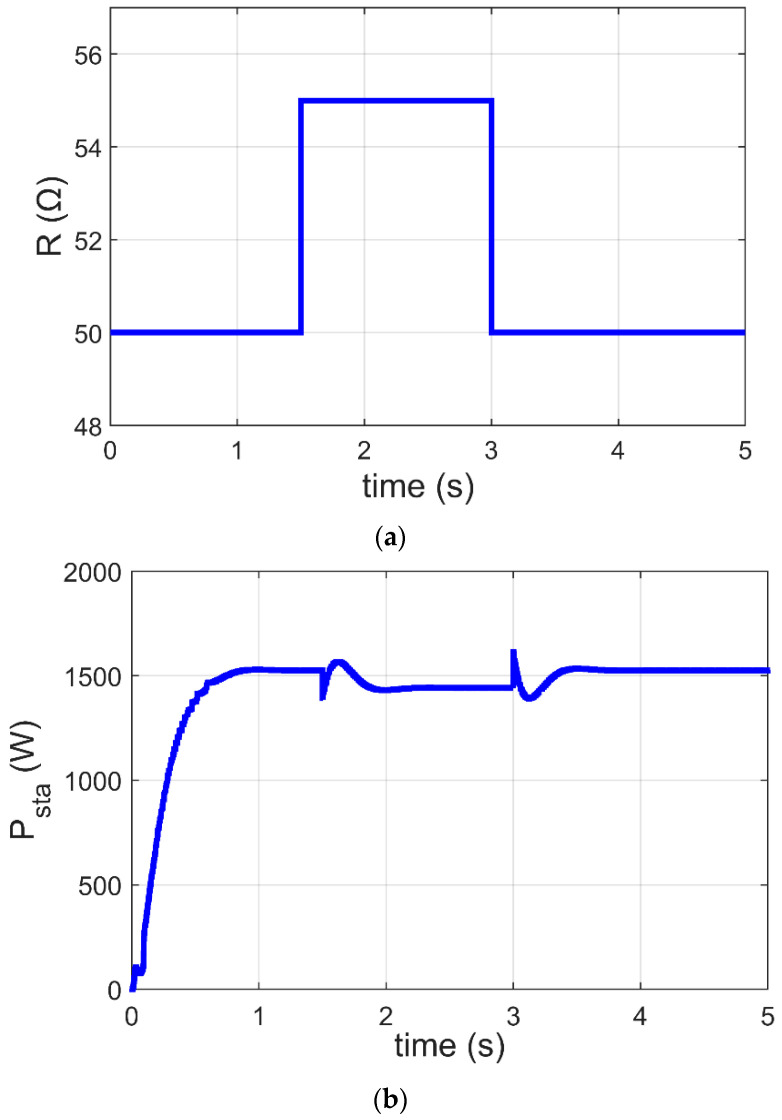
Response of MPPT based on GJOA-PI-PD controller with variation of R. (**a**) Variation of $T_{fc}$. (**b**) P_sta_ of Ballard Mark V PEM-FC.

In this subsection, the GJOA-PI-PD controller for MPPT of the Ballard Mark V PEM-FC is justified when R changes. Figure 13a reveals that the value of R is initially 50 Ω, then it increases to 55 Ω at t=1.5 s and afterward decreases to 50 Ω at t=3 s. Figure 13b reveals the corresponding response of P_sta_ during change in $P_{H_{2}},$ where MPPT based on the GJOA-PI-PD controller responds quickly to variation in $P_{H_{2}}$. During the period of increase in R, P_sta_ decreases to its new value then increases with decrease of R. This points out that P_sta_ tracks new MPP for new conditions. The new conditions in this case study result in variation of R from 50 Ω to 55 Ω and then from 55 Ω to 50 Ω, with constant values of $P_{H_{2}}=1\;atm$ and $T_{fc}=343\;K$. In addition, the oscillations are low.

## 7. Conclusions

The I/P plot of PEM-FC varies with the operating conditions, namely $P_{H_{2}}$, $T_{fc}$, and loading. Accordingly, each group of conditions has a unique I/P plot with unique MPP. Therefore, the presence of the MPPT scheme is required to track MPP continuously. In this work, an innovative MPPT scheme for PEM-FC based on the PI-PD controller, whose gains are optimized via GJOA, has been suggested. The simulation results of the MPPT scheme based on the GJOA-PI-PD controller have been compared with those of other schemes, namely P&O, GJOA-PID, GJOA-FOPID controllers, at normal operating conditions of PEM-FC. The comparison has revealed that the ITAE which resulted using the MPPT scheme based on the GJOA-PI-PD controller is less than that of the compared schemes by 93.95%, 87.17%, and 87.97%, respectively. In addition, the simulation results have revealed that the response of the suggested scheme has the lowest oscillations and overshoot. Furthermore, the MPPT scheme based on the GJOA-PI-PD controller has been legitimized during variation in operating conditions. The simulation results of the MPPT scheme based on the GJOA-PI-PD controller during variation of $P_{H_{2}}$, $T_{fc}$, and loading reveal the high speed of performance. Our research plan in the future is to experimentally legalize the suggested MPPT controller of PEM-FC.

## Figures and Tables

**Table 1:** Brief of limitations of some techniques utilized for MPPT of PEM-FC.

Reference	Technique	Limitations
[12,13,14]	P&O	MPP is not guaranteed due to fluctuations
[15,19,20,21]	IC	Producing error tracking particularly during rapid variation of operating conditions
[16]	IR	Tracking capability is weak during rapid variation of operating conditions
[17,18]	Backstepping	Finest performance is not guaranteed, particularly through system uncertainties
[22,23,24,25,26,27,28]	ANN	Much data is needed for training ANN
[29,30,31]	FL	Responses contain oscillations and large overshoot
[32]	MPC	Responses contain large overshoot
[33,34,35]	SMC	MPP is not guaranteed because of reliance on the sliding surface
[36,37,38,39,40,41,42,43,44,45]	FLC	Responses contain oscillations
[46,47,48]	PID controller	Slow response
[49,50]	FOPID controller	Much effort in execution is needed

**Table 2:** Parameters of Ballard Mark V.

Parameter	Value
$n_{cells}$	35
$\xi_{1}$	−1.1978
$\xi_{2}$	4.4183 × 10^−3^
$\xi_{3}$	9.7214 × 10^−5^
$\xi_{4}$	−16.273 × 10^−5^
$R_{C}\;(m\Omega )$	0.1002
b	0.0136
$J_{max}\;(A/{cm}^{2})$	1.5

**Table 3:** The bounds of GJOA-PID controller parameters.

Parameter	Upper Bound	Lower Bound
$K_{p},K_{i},K_{d}$	0	10

**Table 4:** The bounds of GJOA-FOPID controller parameters.

Parameter	Upper Bound	Lower Bound
$K_{p},K_{i},K_{d}$	0	10
λ	0.1	2
µ	0.1	2

**Table 5:** The bounds of GJOA-PI-PD controller parameters.

Parameter	Upper Bound	Lower Bound
$K_{p_{1}},K_{i},K_{p_{2}},K_{d}$	0	10

**Table 6:** The optimized values of GJOA-PID controller parameters.

Parameter	Value
$K_{p}$	1.1302
$K_{i}$	5.4400
$K_{d}$	0.0468

**Table 7:** The optimized values of GJOA-FOPID controller parameters.

Parameter	Value
$K_{p}$	4.5910
$K_{i}$	7.9302
$K_{d}$	5.3335
λ	0.6794
µ	0.1126

**Table 8:** The optimized values of GJOA-PI-PD controller parameters.

Parameter	Value
$K_{p_{1}}$	10.0000
$K_{i}$	8.7551
$K_{p_{2}}$	10.0000
$K_{d}$	10.0000

**Table 9:** The optimized values of GJOA-FOPID controller parameters.

MPPT Scheme	t_r_ (s)	POS (%)
P&O	0.167	11.5
GJOA-PID Controller	0.545	1.46
GJOA-FOPID Controller	0.483	1.83
GJOA-PI-PD Controller	0.391	0.2

**Table 10:** The values of the resultant ITAE.

MPPT Scheme	ITAE
P&O scheme	0.6597
GJOA-PID controller	0.3109
GJOA-FOPID controller	0.3317
GJOA-PI-PD controller	0.0399

## Data Availability

Not applicable.
